# Ideal Cardiovascular Health Metrics Modify the Association Between Exposure to Chinese Famine in Fetal and Cardiovascular Disease: A Prospective Cohort Study

**DOI:** 10.3389/fcvm.2021.751910

**Published:** 2021-11-04

**Authors:** Xiong Ding, Jinfeng Li, Ying Wu, Peng Yang, Dandan Zhao, Xiaojie Yuan, Shuohua Chen, Xiaoyan Luo, Yun Li, Shouling Wu

**Affiliations:** ^1^School of Public Health, North China University of Science and Technology, Tangshan, China; ^2^Department of Cardiology, Kailuan General Hospital, Tangshan, China; ^3^Department of Neurosurgery, Affiliated Hospital of North China University of Science and Technology, Tangshan, China; ^4^Department of Epidemiology and Biostatistics, Institute of Basic Medical Sciences Chinese Academy of Medical Sciences, School of Basic Medicine Peking Union Medical College, Beijing, China; ^5^Department of Emergency, Kailuan General Hospital, Tangshan, China

**Keywords:** fetal, ideal cardiovascular health metrics, China famine, cardiovascular disease, cohort study

## Abstract

**Background:** No study has explored the modification effect of ideal cardiovascular health metrics (ICVHMs) on the association between famine exposure and risk of cardiovascular disease (CVD) so far. We aim to examine the effect of ICVHMs on the association between exposure to famine early in life and the risk of CVD in adulthood.

**Methods:** A total of 61,527 participants free of CVD were included in this study from the Kailuan Study. All participants were divided into three groups, included nonexposed, fetal-exposed, and childhood-exposed groups. Cox regression was used to estimate the effect of famine exposure and ICVHMs on CVD risk.

**Results:** After a median of 13.0 (12.7–13.2) years follow-up, 4,814 incident CVD cases were identified. Compared with nonexposed participants, the CVD risk increased in participants with fetal famine exposure (hazard ratio [HR]: 1.21; 95% CI: 1.07–1.37), but not in childhood famine-exposed participants. After stratifying by the number of ICVHMs, the increased CVD risk associated with fetal famine exposure was only observed in participants with less ICVHMs ( ≤ 2) (HR: 1.30; 95% CI: 1.11–1.52, *P* for interaction=0.008), but disappeared in those with three or more ICVHMs. The modified effect of ICVHMs was sex specific (*P* for sex interaction = 0.031).

**Conclusions:** Exposing to famine in the fetal period could increase the risk of CVD in late life; however, ICVHMs might modify the effect of famine exposure on CVD risk, especially in men.

## Introduction

Cardiovascular disease (CVD), the most common noncommunicable disease and the leading cause of mortality globally, is an important contributor to the disease burden (1). Reducing the incidence of CVD will be helpful in lowering the burden of this disease and promoting health. Previous studies have demonstrated an association between famine exposure in early life and health effects in adulthood, such as hypertension (2), diabetes mellitus (3), stroke (4, 5), myocardial infarction (MI) (4), and metabolic syndrome (4, 6). The Great Chinese Famine of 1959–1962 was one of the largest famines in human history (7), swiped almost all of the mainland of China. Individuals who were born around the period experienced various severity of famine in early life and were the high-risk populations of CVD. Modifying or reversing the effect of famine exposure on CVD risk might help to reduce the incidence of CVD in these high-risk populations.

Ideal cardiovascular health metrics (ICVHMs) were first proposed in 2010 by the American Heart Association (8), which consisted of seven items, four behavioral metrics (nonsmoking, ideal body mass index [BMI], physical activity at goal levels, and a dietary pattern recommended) and three biological metrics (ideal levels of untreated blood pressure [BP], fasting blood glucose [FBG], and total cholesterol [TC]). Accumulating epidemiological evidence suggested that higher ICVHMs were associated with a lower risk of CVD (9), and all-cause death (10, 11). In 2020, Lu et al. (12) reported that ICVHMs might modify the association between famine exposure in early life and the risk of diabetes mellitus. However, to the best of our knowledge, there have been no studies to explore whether ICVHMs in later life modify the association between famine exposure and risks of CVD in adulthood. We conducted this prospective study in the Kailuan Study, an ongoing cohort enrolled approximately 100,000 participants beginning in 2006, with aims to examine the association between exposure to the Great Chinese Famine in early life and risk of CVD in adults and the modification effect of ICVHMs.

## Methods

### Study Design and Population

The Kailuan Study (trial registration number ChiCTR-TNC-11001489) is an ongoing longitudinal prospective study conducted in the Kailuan community in Tangshan, Republic of China. A total of 101,510 employees (including the retired, 81,110 men and 20,400 women, mean age = 51.9 y in 2006) of the Kailuan Group were invited and agreed to participate in the Kailuan Study between June 2006 and October 2007 (referred to as the “baseline survey”). Detailed study design and procedures have been described in previous studies (13, 14). All participants completed questionnaire assessments, clinical examinations, and laboratory tests in 11 hospitals responsible for the health care of the Kailuan general hospital. Participants then took physical examination every 2 years, and the incidences of chronic diseases (e.g., stroke, MI, cancer, et al.) were recorded annually. In the current study, data analysis was performed from baseline survey to December 31, 2019.

In the present study, according to previous Chinese famine studies (4, 12), 65,105 participants born between January 1, 1949, and December 31, 1974, were recruited from the Kailuan Study. Participants (n = 1,060) who had a history of MI or stroke at the baseline survey were excluded. Moreover, we excluded participants (n = 2,518) who had incomplete data on health factors or behaviors, leaving 61,527 participants (47,549 men and 13,978 women) available for analyses (Supplementary Figure S1).

### Data Collection

We collected information on sociodemographic characteristics (e.g., age, gender, birth date, and education level), lifestyle factors (e.g., smoking, alcohol intake, salt intake, and physical activity), medical history (e.g., CVD, hypertension, diabetes mellitus, family history of CVD), and active treatments such as hypoglycemic, antihypertensive, and lipid-lowering medications through the self-reported questionnaire in the Kailuan Study since the baseline survey, as detailed previously (13). Education was classified as less than high school and high school or above. Drinking status was stratified into two levels: current or never/former. Weight and height were measured, and BMI was calculated as weight (kg)/height (m)^2^. Moreover, BP was measured on the left arm using an appropriate cuff size after the participant had a rest in a chair for at least 5 min. The average values of at least two readings each of systolic and diastolic BPs were used for further analysis.

The blood sample of each participant was collected on the morning of the survey after at least a 12-h fast. All samples were measured by a Hitachi 747 autoanalyzer at the central laboratory of the Kailuan General Hospital. The estimated glomerular filtration rate (eGFR) was calculated using the Chronic Kidney Disease Epidemiology Collaboration creatinine equation (15).

### Famine Exposure and Severity

Because the Chinese Famine occurred from 1959 to 1962, consistent with previous Chinese famine studies (4, 12, 16, 17), birth year was taken as the proxy variable of exposure to famine in this study. We defined those born between January 1, 1959, and December 31, 1962, as fetal exposure group, those born between January 1, 1949, and December 31, 1958, as childhood-exposed group, and those born between January 1, 1963, and December 31, 1974, as a nonexposed group. As a large enterprise group, the employees in the Kailuan group were nationwide. Therefore, the severity of the famine was determined according to the excess death rate for each province, which was calculated as this rate change from the average level in 1956–1958 to the highest value in 1959–1962 (7). Based on the information, an excess mortality rate of 50% was used as a threshold value to define severely and less severely affected areas in the present study.

### Ascertainment of ICVHMs Metrics

ICVHMs were adapted from the recommendations of American Heart Association (8): nonsmoking, BMI <24 kg/m^2^, physical activity at goal (at least 80 min per week of moderate-intensity physical activity), ideal diet (daily salt intake <6 g) (18); TC <5.2 mmol/L (untreated), BP <120/80 mmHg (untreated), FBG <5.6 mmol/L (untreated). Cardiovascular health status was then categorized as high (5–7 metrics), moderate (3–4 metrics), or low (0–2 metrics) levels (19).

### Ascertainment of Incident CVD Events

The main outcome of this study was the first occurrence of CVD events, including MI, severe coronary artery disease, intracerebral hemorrhagic stroke, and ischemic stroke. CVD events were defined as described (20, 21). Briefly, the Municipal Social Insurance Institution database and Hospital Discharge Register were linked to identify the incidence of CVD based on *The International Statistical Classification of Diseases and Related Health Problems 10th Revision* (ICD-10) (I61 for intracerebral hemorrhagic stroke, I63 for ischemic stroke, and I21 for MI, I25.1 for coronary heart disease). These two databases covered all the Kailuan Study participants. For all suspected CVD cases, a panel of three experienced physicians, consisting of neurologists, cardiologists, and radiologists, reviewed the medical records, blind to exposure status. We defined coronary artery disease and MI cases according to the Multinational Monitoring of Trends and Determinants in CVD criteria of WHO on basis of clinical symptoms and dynamic changes in cardiac enzymes and/or biomarker concentrations and electrocardiogram results. Severe coronary artery disease was defined as coronary heart disease treated with coronary bypass surgery or stent placement. Incident stroke was diagnosed according to the WHO criteria, based on symptoms, neuroimages (from CT or MRI), and other diagnostic reports. Mortality data were collected from provincial vital statistics offices, as described previously (14).

### Statistical Analyses

All analyses were performed using SAS, version 9.4 (SAS Institute, Inc, Cary, NC). Two-sided values of *P* < 0.05 were regarded as significant.

The baseline characteristics of the study population were described by famine exposure status. Continuous variables with normal distribution were expressed as means ± SDs and compared using one-way ANOVA analysis, while those with skewed distribution were expressed as medians and interquartile range and compared by Kruskal-Wallis test. Categorical variables were shown in proportions and compared by Pearson's Chi-Square test.

We computed the person-year of follow-up for each participant from the date of the baseline survey to the date of the CVD onset, death, loss to follow-up (n=2,156, 3.5%), or the end of follow-up (December 31, 2019), whichever came first. The Cox regression model was used to predict the risk of CVD and to estimate the hazard ratio (HR) and 95% CI, after adjustments for age, sex, education attainment (less than high school, high school, or above), drinking (current, never/former), eGFR (<30, 30 ≤ eGFR <60, or ≥ 60 mL/min/1.73 m^2^), and high-sensitivity C-reactive protein (Hs-CRP) (<1.0, 1.0 ≤ Hs-CRP ≤ 3.0, or > 3.0 mg/L), triglycerides (TG) (<1.7, 1.7 ≤ TG <2.3, or ≥ 2.3 mmol/L), severity famine exposed, family history of CVD, using of antihypertensive, hypoglycemic, lipid-lowering medications (yes/no for each), and individual ICVHMs. Cochran-Armitage trend test was used to investigate the association between the increasing number of ICVHMs and the cumulative incidence of CVD. Missing covariates (drinking, eGFR, Hs-CRP, TG) will be imputed by means of multiple imputations using the Fully Conditional Specification method computing 10 imputed datasets and to prevent case-wise deletion of missing data; 60,874 (98.9%) participants had complete data for all covariates.

To explore the modifying effect of ICVHMs, we evaluated the association between famine exposure and CVD by strata of the individual ICVHMs items and the number of ICVHMs. Other ICVHMs items were adjusted when evaluating individual ICVHMs items. *P*-value was corrected for multiple testing *via* false discovery rate using the Benjamini-Hochberg method. To demonstrate potential interactions of famine exposure and ICVHMs on the development of CVD, we generated interaction terms using the cross products of famine exposure and ICVHMs in Cox regression models. To address specific questions on age differences between famine and postfamine births, an “age-balanced” method was used, in which both prefamine and postfamine births were combined as unexposed control subjects, as detailed previously (12, 22).

We further conducted several sensitivity analyses. To assess the impact of imputing missing covariates, we repeated the main analyses in those without imputation. To test the robustness of our finding, we repeated the main analyses excluding those with antihypertensive, hypoglycemic, or lipid-lowering medications treatment, excluding those lost to follow-up, and excluding those who had CVD events or death within the first 2 years of follow-up.

## Results

### Participant Characteristics

A total of 61,527 participants (mean age 47.9 ± 6.6 y) were recruited in the present analyses, including 47,549 (77.3%) men and 13,978 (22.7%) women. There were 13.9% (n = 8,572), who were exposed to the Great Chinese Famine in the fetal stage. Compared with the nonexposed group (born between January 1, 1963, and December 31, 1974), participants with fetal famine exposure were more likely to be with a family history of CVD, or use antihypertensives or hypoglycemics, had higher BMI, TC, TG, systolic or diastolic BPs, FBG level, Hs-CRP level, had a greater proportion of individuals with ideal physical activity, as well as lower eGFR, education level, less proportion of individuals with other six individual ideal items, and less drinkers (Table 1). Supplementary Table S1 showed baseline information for the age-balanced group with combining of prefamine and postfamine births as the reference group.

**Table 1:** Baseline characteristics of participants according to famine exposure in early life.

	**Nonexposed**	**Famine exposure**		***P* value**
		**Fetal**	**Childhood**	
Number of participants	18,761	8,572	34,194	
Age at baseline, years	39.6 ± 3.4	46.1 ± 1.3	52.8 ± 2.8	<0.001
Men	13,844 (73.8)	6,271 (73.2)	27,434 (80.2)	<0.001
BMI, kg/m^2^	25.0 ± 3.5	25.1 ± 3.4	25.2 ± 3.3	<0.001
High school education or above	5,142 (27.4)	1,517 (17.7)	3,999 (11.7)	<0.001
Current drinking	8,388 (44.7)	3,508 (40.9)	13,652 (39.9)	<0.001
TC, mmol/L	4.9 ± 1.1	5.0 ± 1.1	5.0 ± 1.1	<0.001
TG, mmol/L	1.3 (0.9, 2.0)	1.3 (0.9,2.0)	1.2 (0.9, 2.0)	<0.001
SBP, mmHg	123.3 ± 17.7	127.4 ± 19.1	131.9 ± 20.4	<0.001
DBP, mmHg	81.8 ± 11.8	83.7 ± 12.0	84.9 ± 11.9	<0.001
FBG, mmol/L	5.3 ± 1.3	5.5 ± 1.7	5.6 ± 1.7	<0.001
Hs-CRP, mg/L	0.6 (0.2, 1.5)	0.6 (0.2,1.6)	0.8 (0.3, 2.1)	<0.001
eGFR, ml/min/1.73m^2^	87.9 (73.7, 105.4)	83.4 (70.3,99.2)	80.7 (68.3–95.5)	<0.001
Family history of CVD	1,288 (6.9)	603 (7.0)	2,325 (6.8)	0.741
Severity famine exposed	1,156 (6.2)	665 (7.8)	2,320 (6.8)	<0.001
Use of antihypertensive agent	779 (4.2)	583 (6.8)	3,723 (10.9)	<0.001
Use of hypoglycemic medications	121 (0.6)	118 (1.4)	766 (2.2)	<0.001
Use of lipid-lowering medications	82 (0.4)	35 (0.4)	274 (0.8)	<0.001
Ideal BP	5,486 (29.2)	1,868 (21.8)	5,709 (16.7)	<0.001
Ideal FBG	13,966 (74.4)	5,947 (69.4)	22,539 (65.9)	<0.001
Ideal TC	12,254 (65.3)	5,006 (58.4)	19,324 (56.5)	<0.001
Ideal BMI	7,711 (41,1)	3,364 (39.2)	12,643 (37.0)	<0.001
Ideal smoking	11,333 (60,4)	5,129 (59.8)	20,107 (58.8)	0.001
Ideal salt intake	1,705 (9.1)	701 (8.2)	2,963 (8.7)	0.039
Ideal physical activity	1,145 (6.1)	625 (7.3)	4,859 (14.2)	<0.001
No. of ICVHMs				<0.001
≤ 2	7,312 (39.0)	3,968 (46.3)	16,403 (48.0)	
3–4	9,481 (50.5)	4,059 (47.4)	15,926 (46.6)	
≥5	1,968 (10.5)	545 (6.3)	1,865 (5.4)	

*BMI, body mass index; TC, total cholesterol; TG, triglycerides; SBP, systolic blood pressure; DBP, diastolic blood pressure; FBG, fasting blood glucose; Hs-CRP, high-sensitivity C-reactive protein; eGFR, estimated glomerular filtration rate; BP, blood pressure; ICVHMs, ideal cardiovascular health metrics*.

*Data were present as n (%), mean ± SD, or median (P_25_, P_75_) according to variable category. Pearson's chi-square test, ANOVA analysis, or Kruskal-Wallis test was used to compare differences between groups properly*.

### Association Between Famine Exposure and CVD

During a median of 13.0 (12.7–13.2) years follow-up, 4,814 incident CVD events were identified. Age of CVD onset was 49.5±4.3, 54.2±3.8, 60.8±4.4 for the nonexposed, fetal-exposed, or childhood-exposed group, respectively (Supplementary Table S2). As shown in Table 2, both the incidences of CVD for fetal famine-exposed (5.95/1000 pys) or childhood famine-exposed (8.31/1000 pys) group were greater than that for the nonexposed group (3.10/1000 pys). After adjusting for covariates, the increased CVD risk remained significant in fetal famine-exposed participants (HR: 1.21; 95% CI: 1.07–1.37), but not in childhood famine-exposed participants (HR: 1.00; 95% CI: 0.85–1.17). Furthermore, in the sex stratified analysis (Table 2) and age-balanced analysis (Supplementary Table S3), the results were similar.

**Table 2:** HR (95% CI) for incident CVD according to famine exposure in early life.

	**Nonexposed**	**Famine exposure**	
		**Fetal**	**Childhood**
Total			
Case subjects/total number	737/18,761	632/8,572	3,445/34,194
IR, 1000 person-years	3.10 (2.89–3.34)	5.95 (5.51–6.44)	8.31 (8.04–8.59)
Univariate model	1.00 (Reference)	1.93 (1.73–2.14)	2.71 (2.50–2.94)
Age- and sex- adjusted model	1.00 (Reference)	1.25 (1.11–1.42)	1.04 (0.88–1.21)
Multivariate model 1	1.00 (Reference)	1.27 (1.12–1.43)	1.05 (0.89–1.23)
Multivariate model 2	1.00 (Reference)	1.21 (1.07–1.37)	1.00 (0.85–1.17)
Sex P for interaction=0.051			
Men			
Case subjects/total number	670/13,844	541/6,271	3,061/27,434
IR, 1,000 person-years	3.83 (3.55–4.14)	7.00 (6.43–7.61)	9.29 (8.97–9.62)
Multivariate model	1.00 (Reference)	1.16 (1.02–1.33)	0.98 (0.83–1.15)
Women			
Case subjects/total number	67/4,917	91/2,301	384/6,760
IR, 1,000 person-years	1.07 (0.84–1.36)	3.15 (2.57–3.87)	4.52 (4.09–4.99)
Multivariate model	1.00 (Reference)	1.67 (1.17–2.40)	1.21 (0.74–1.96)

*HR, hazard ratio; CI, confidence interval; CVD, cardiovascular disease; IR, incidence rate*.

*Multivariate model 1: Adjusted for age, sex, education attainment (less than high school, high school, or above), drinking (current, never/former), Hs-CRP (<1.0, 1.0 ≤ Hs-CRP ≤ 3.0, or >3.0 mg/L), eGFR (<30, 30 ≤ eGFR <60, or ≥60 ml/min/1.73m^2^), and TG (<1.7, 1.7 ≤ TG <2.3, or ≥ 2.3 mmol/L)*.

*Multivariate model 2: Included covariates in multivariate model 1 and further adjusted for the severity of famine exposed, family history of CVD, use of antihypertensive, hypoglycemic, and lipid-lowering medications (yes/no for each), and individual ideal cardiovascular health metrics*.

*Multivariate model in men or women: Adjusted for age, education attainment (less than high school, high school, or above), drinking (current, never/former), Hs-CRP (<1.0, 1.0 ≤ Hs-CRP ≤ 3.0, or > 3.0 mg/L), eGFR (<30, 30 ≤ eGFR <60, or ≥60 ml/min/1.73m^2^), TG (<1.7, 1.7 ≤ TG <2.3, or ≥ 2.3 mmol/L), severity famine exposed, family history of CVD, use of antihypertensive, hypoglycemic, and lipid-lowering medications (yes/no for each) and individual ideal cardiovascular health metrics*.

### Modified Effect of ICVHMs on the Association Between Famine Exposure and CVD

**Figure 1:**
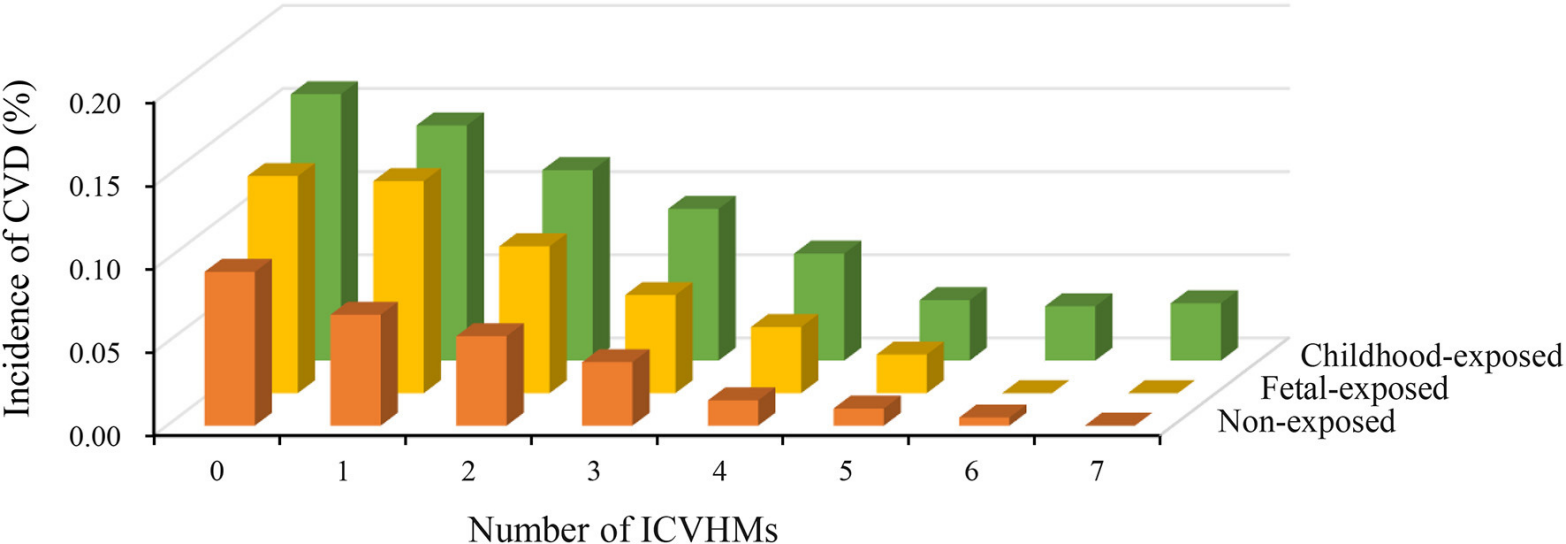
Cumulative incidence of CVD according to famine exposure and the number of ICVHMs.

The incidence of CVD according to famine exposure and the number of ICVHMs were displayed in Figure 1. There was a reverse correlation between the number of ICVHMs and the incidence of CVD. With the increasing number of ICVHMs, the cumulative incidence of CVD decreased gradually for participants in nonexposed or fetal-exposed groups (all *P* for trend < 0.001).

After stratifying participants by the number of ICVHMs, we found that in participants with less ICVHMs ( ≤ 2), fetal famine exposure significantly increased the CVD risk after adjusting for all covariates (HR: 1.30; 95% CI: 1.11–1.52, *P* for interaction=0.008), comparing with a nonexposed group (Table 3). However, we do not observe the association in participants with three or more ICVHMs (HR: 1.14; 95% CI: 0.92–1.40, HR: 0.89; CI: 0.37–2.11). In sensitivity analyses, consistent results were observed without imputing covariates, excluding participants using antihypertensive, hypoglycemic, or lipid-lowering medications, participants lost to follow-up, and who had events or death within 2 years of follow-up (Table 3). Similar results were observed in the age-balanced analysis (Supplementary Table S4).

**Table 3:** Multivariable adjusted HR (95% CI) for incident CVD according to famine exposure and combined ICVHMs.

	**Case subjects/total number**	**IR, 1,000 person-years**	**Nonexposed**	**Famine exposure**		***P* for interaction**
				**Fetal**	**Childhood**	
No. of ICVHMs						0.008
≤ 2	2,933/27,683	8.73 (8.42–9.05)	1.00 (Reference)	1.30 (1.11–1.52)	1.12 (0.92–1.37)	
3–4	1,784/29,466	4.87 (4.65–5.10)	1.00 (Reference)	1.14 (0.92–1.40)	0.93 (0.72–1.21)	
≥5	97/4,378	1.75 (1.43–2.13)	1.00 (Reference)	0.89 (0.37–2.11)	0.51 (0.16–1.60)	
Sensitivity analyses						
Without imputation						
No. of ICVHMs						0.008
≤ 2	2,933/27,683	8.73 (8.42–9.05)	1.00 (Reference)	1.30 (1.11–1.52)	1.12 (0.92–1.37)	
3–4	1,784/29,466	4.87 (4.65–5.10)	1.00 (Reference)	1.14 (0.92–1.40)	0.93 (0.72–1.21)	
≥5	97/4,378	1.75 (1.43–2.13)	1.00 (Reference)	0.88 (0.37–2.11)	0.51 (0.16–1.60)	
Excluding 5,873 participants antihypertensive, hypoglycemic, or lipid-lowering medications users						
No. of ICVHMs						0.049
≤ 2	2,223/23,631	7.69 (7.37–8.01)	1.00 (Reference)	1.27 (1.07–1.51)	1.11 (0.88–1.40)	
3–4	1,558/27,738	4.50 (4.28–4.73)	1.00 (Reference)	1.08 (0.87–1.34)	0.84 (0.63–1.10)	
≥5	92/4,285	1.69 (1.38–2.08)	1.00 (Reference)	0.89 (0.37–2.12)	0.48 (0.15–1.52)	
Excluding 2,156 participants lost to follow up						
No. of ICVHMs						0.007
≤ 2	2,933/26,707	9.07 (8.75–9.41)	1.00 (Reference)	1.29 (1.10–1.50)	1.11 (0.91–1.36)	
3–4	1,784/28,423	5.06 (4.83–5.30)	1.00 (Reference)	1.12 (0.91–1.38)	0.92 (0.71–1.20)	
≥5	97/4,241	1.80 (1.48–2.20)	1.00 (Reference)	0.88 (0.37–2.09)	0.50 (0.16–1.57)	
Excluding 321 participants who had events or death within 2 years of follow-up						
No. of ICVHMs						0.010
≤ 2	2,872/27,512	8.55 (8.24–8.87)	1.00 (Reference)	1.30 (1.11–1.52)	1.14 (0.93–1.39)	
3–4	1,750/29,327	4.78 (4.56–5.01)	1.00 (Reference)	1.15 (0.94–1.42)	0.96 (0.73–1.25)	
≥5	95/4,367	1.71 (1.40–2.09)	1.00 (Reference)	0.87 (0.37–2.09)	0.47 (0.15–1.50)	

*HR, hazard ratio; CI, confidence interval; CVD, cardiovascular disease; ICVHMs, ideal cardiovascular health metrics; IR, incidence rate*.

*Adjusted for age, sex, education attainment (less than high school, high school, or above), drinking (current, never/former), Hs-CRP (<1.0, 1.0 ≤ Hs-CRP ≤ 3.0, or > 3.0 mg/L), eGFR (<30, 30 ≤ eGFR <60, or ≥60 ml/min/1.73m^2^), TG (<1.7, 1.7 ≤ TG <2.3, or ≥ 2.3 mmol/L), severity famine exposed, family history of CVD, use of antihypertensive, hypoglycemic, and lipid-lowering medications (yes/no for each)*.

The sex difference was further explored in stratified analysis. Due to the small sample size of participants with ≥5 ICVHMs items in men or women, we combined and divided men or women into two groups according to the number of ICVHMs: participants with less ICVHMs ( ≤ 2) items or more ICVHMs (≥3) items. Similar results were observed in men, that the association between fetal famine exposure and CVD risk was significant in men with less ICVHMs (HR: 1.26, 95% CI: 1.07–1.49) rather than those with three or more ICVHMs (HR: 1.05, 95% CI: 0.84–1.31). However, results were inverse in women, that the association between fetal famine exposure and CVD risk was significant in women with three or more ICVHMs (HR: 1.65, 95% CI: 1.01–2.69) rather than those with less ICVHMs (HR: 1.58, 95% CI: 0.92–2.70). The interaction between sex and famine exposure was significant (*P* = 0.031) (Table 4).

**Table 4:** Multivariable adjusted HR (95% CI) for CVD according to sex and famine exposure.

	**Case subjects/total number**	**IR, 1,000 person years**	**Nonexposed**	**Famine exposure**		*P* for interaction^*^
				**Fetal**	**Childhood**	
Sex (men vs. women)						0.031
Men						
Number of ICVHMs						
≤ 2	2,696/24,416	9.12 (8.78–9.47)	1.00 (Reference)	1.26 (1.07–1.49)	1.14 (0.93–1.41)	
≥3	1,576/23,133	5.51 (5.24–5.79)	1.00 (Reference)	1.05 (0.84–1.31)	0.81 (0.61–1.08)	
Women						
Number of ICVHMs						
≤ 2	237/3,267	5.85 (5.15–6.64)	1.00 (Reference)	1.58 (0.92–2.70)	0.83 (0.39–1.76)	
≥3	305/10,711	2.24 (2.01–2.51)	1.00 (Reference)	1.65 (1.01–2.69)	1.51 (0.79–2.86)	

*HR, hazard ratio; CI, confidence interval; CVD, cardiovascular disease; IR, incidence rate; ICVHMs, ideal cardiovascular health metrics*.

*Adjusted for age, education attainment (less than high school, high school, or above), drinking (current, never/former), Hs-CRP (<1.0, 1.0 ≤ Hs-CRP ≤ 3.0, or > 3.0 mg/L), eGFR (<30, 30 ≤ eGFR <60, or ≥60 ml/min/1.73m^2^), TG (<1.7, 1.7 ≤ TG <2.3, or ≥ 2.3 mmol/L), severity famine exposed, family history of CVD, use of antihypertensive, hypoglycemic, and lipid-lowering medications (yes/no for each)*.

* *P for interaction was between sex and famine exposure group*.

We further analyzed the association between famine exposure and CVD risk according to individual ICVHMs items. The associations between famine exposure and CVD risk were observed in participants with nonideal ICVHMs items, except for TC (in ideal TC) and smoking items (in both ideal and nonideal) (Table 5). Multiple testing *via* false discovery rate analyses showed similar results (Supplementary Table S5). In age-balanced analysis, the risk estimates did not change dramatically (Supplementary Table S6).

**Table 5:** Multivariable adjusted HR (95% CI) for incident CVD according to famine exposure and individual ICVHMs items.

	**Case subjects/total number**	**IR, 1,000 person-years**	**Nonexposed**	**Famine exposure**	
				**Fetal**	**Childhood**
BP					
Ideal	473/13,063	2.87 (2.62–3.14)	1.00 (Reference)	1.31 (0.91–1.91)	1.01 (0.61–1.67)
Nonideal	4,341/48,464	7.32 (7.10–7.54)	1.00 (Reference)	1.21 (1.06–1.37)	1.01 (0.85–1.19)
FBG					
Ideal	2,832/42,452	5.37 (5.18–5.57)	1.00 (Reference)	1.17 (0.99–1.37)	0.98 (0.80–1.20)
Nonideal	1,982/19,075	8.58 (8.21–8.97)	1.00 (Reference)	1.31 (1.08–1.60)	1.08 (0.84–1.39)
TC					
Ideal	2,457/36,584	5.42 (5.21–5.64)	1.00 (Reference)	1.26 (1.06–1.50)	1.02 (0.82–1.28)
Nonideal	2,357/24,943	7.73 (7.42–8.05)	1.00 (Reference)	1.18 (0.99–1.40)	1.01 (0.81–1.27)
BMI					
Ideal	1,353/23,718	4.59 (4.35–4.84)	1.00 (Reference)	1.11 (0.88–1.41)	0.92 (0.68–1.25)
Nonideal	3,461/37,809	7.47 (7.22–7.72)	1.00 (Reference)	1.28 (1.10–1.47)	1.06 (0.88–1.28)
Smoking					
Ideal	2,495/36,569	5.51 (5.30–5.73)	1.00 (Reference)	1.22 (1.03–1.45)	0.94 (0.75–1.18)
Nonideal	2,319/24,958	7.59 (7.29–7.91)	1.00 (Reference)	1.24 (1.04–1.48)	1.11 (0.89–1.39)
Salt intake					
Ideal	395/5,369	5.93 (5.38–6.55)	1.00 (Reference)	1.15 (0.73–1.79)	1.11 (0.63–1.94)
Nonideal	4,419/56,158	6.39 (6.21–6.58)	1.00 (Reference)	1.23 (1.08–1.40)	1.01 (0.86–1.19)
Physical activity				
Ideal	563/6,629	6.91 (6.36–7.51)	1.00 (Reference)	1.10 (0.69–1.74)	0.95 (0.55–1.64)
Nonideal	4,251/54,898	6.28 (6.10–6.47)	1.00 (Reference)	1.23 (1.08–1.40)	1.01 (0.86–1.20)

*HR, hazard ratio; CI, confidence interval; CVD, cardiovascular disease; ICVHMs, ideal cardiovascular health metrics; IR, incidence rate; BP, blood pressure; FBG, fasting blood glucose; TC, total cholesterol; BMI, body mass index*.

*Adjusted for age, sex, education attainment (less than high school, high school, or above), drinking (current, never/former), Hs-CRP (<1.0, 1.0 ≤ Hs-CRP ≤ 3.0, or > 3.0 mg/L), eGFR (<30, 30 ≤ eGFR <60, or ≥60 ml/min/1.73m^2^), TG (<1.7, 1.7 ≤ TG <2.3, or ≥ 2.3 mmol/L), severity famine exposed, family history of CVD, use of antihypertensive, hypoglycemic, and lipid-lowering medications (yes/no for each), and individual ideal cardiovascular health metrics were mutually adjusted*.

## Discussions

In this large community-based prospective study, we observed that fetal exposure to the Great Chinese Famine was associated with an increased risk of CVD in adults. More importantly, ICVHMs may modify the association between fetal exposure to famine and the risk of CVD in adults. The effect of fetal exposure to famine on the CVD risk only occurred in participants with less ICVHMs items but not in those with more ICVHMs items. The results not only emphasized the importance of adequate nutrition in early life but also suggested a healthy lifestyle in late life. The results are of great significance for that experienced famine or malnutrition in early life to reduce the risk of CVD.

Previous epidemiological studies have reported conflicting results regarding the association between famine exposure and CVD (23–25). In a median 10.1 years follow-up of 92,284 participants, in urban areas, compared with nonexposed births, famine births had a higher risk of cerebrovascular disease (HR 1.18; 95% CI: 1.09–1.28) (23). Similarly, in the present study, our results supported the association between famine exposure in early life and CVD risk. However, two studies (24, 25) from the Dutch Famine Birth Cohort have reported that prenatal exposure to the Dutch famine of 1944–1945 was not associated with the subsequent risk of stroke or coronary artery disease in middle-aged adults. Both these two studies were limited with a relatively small sample (~3,000 participants), which might contribute to the different results. In addition, compared with the Dutch famine (~6 months), the Chinese Great Famine lasted longer (~4 years) and had greater severity, affected 600 million population and led to some 30 million premature deaths (7, 26).

There were studies that demonstrated the preventive effect of ICVHMs on CVD risk (19, 27). However, limited researches addressed the modified effect of ICVHMs on the association between famine exposure and CVD risk. In the current study, we examined the effect of ICVHMs on the association between famine exposure in early life and the risk of CVD in a prospective study. We, interestingly, found that the association between famine exposure and CVD risk only showed in participants with less ICVHMs items ( ≤ 2), not in those with ≥3 ICVHMs items, which suggested that the increased risk of CVD owing to famine exposure in early life might be modified by ICVHMs. Individual ICVHMs items also supported the modified effects. There are few studies exploring the modified effect of ICVHMs on the influence of famine exposure. Meng et al. (23) examined physical activity level, one of the ICVHMs items, in 92,284 participants born between 1954 and 1964, and concluded that the increased risk of CVD affected by prenatal famine exposure was only shown in participants with lower physical activity levels, but not in higher ones. Ding et al. (28) demonstrated that a healthy lifestyle, such as adequate physical activity, may partially alleviate the adverse effects of increasing TC in participants with prenatal or early postnatal exposure to famine. Lu et al. (12)reported that ICVHMs might modify the association between famine exposure in early life and the risk of diabetes mellitus.

There are several potential mechanisms by which ICVHMs modify the association between famine exposure in early life and the risk of CVD in adulthood. Stein et al. (29, 30) found that maternal exposure during pregnancy to famine was associated with higher BP, the major risk factor of CVD, in later life. A healthy lifestyle such as moderate physical exercise and a low-salt diet may lower BP (31), and eventually reduced the risk of CVD (32). Fetal exposure to famine was associated with hyperglycemia and diabetes mellitus (3) in adulthood, which could be attenuated by ≥3 ICVHMs (12), and then might reduce the CVD risk. As is well known, higher pulse wave velocity was a risk factor for CVD. The number of ICVHMs items was inversely associated with pulse wave velocity, every one-point increase corresponded to a 0.09-m/s decrease in pulse wave velocity (33), which might be one potential mechanism for our results. Finally, the ICVHMs attained in mid- to late-life could improve the cardiovascular structure and function of blood vessels in late life (34), and subsequently reduce the incidence of CVD.

Sex differences of the associations between early life famine exposure and metabolic diseases were often reported. Similarly, we also observed a sex difference results in the present study. In men, not in women, more ICVHMs items (≥3) might mitigate the effect of fetal famine exposure on the CVD risk (*P* for interaction = 0.031). Evidence showed that male usually was more susceptible to adverse environmental conditions, and female may be more adaptable to famine (35). Therefore, healthy cardiovascular health has a greater effect on male. In addition, due to the traditional Chinese male-sex-preference culture (36), female may suffer from severer famine and have a lower chance to survive than male after birth. But the female survivors might be healthier, and hence be less affected by the environmental conditions, included ICVHMs. Moreover, lots of studies have confirmed the cardiovascular protective effect of estrogen, which might play a stronger effect on cardiovascular health than ICVHMs in female. All these might contribute to the less protective effect of ICVHMs in female.

The strengths of the current study include a prospective cohort design with a large sample size, detailed information about lifestyle factors, and long follow-up time. The diagnosis of CVD was not self-reported but linked to the Municipal Social Insurance Institution database and Hospital Discharge Register for incidence of CVD. However, there were several limitations to our study. First, misclassification of famine exposure may exist, which might underestimate the effects of famine exposure. However, using the birth date of an individual to define famine exposure was the most common method in studies on the Great Chinese Famine (12, 17). Second, the ideal diet calculated by the AHA Diet Score should be calculated on the basis of consumption of whole grains, fruits, vegetables, fish, sodium, sweets, and sugary beverages. Although no detailed dietary composition data were collected in this study, our previous work found a strong association between higher salt intake and lower healthy eating scores. So, we use daily salt intake as a surrogate indicator of diet quality (37). Third, most of the participants were men (77.3%) in the current study; therefore, the observed results may not be generalizable to other populations. However, similar associations between the exposure to famine in early life and risks of CVD have been shown in other nationwide prospective cohort studies, suggesting the broad generalizable nature of the data (4, 23). Finally, although we used age-balanced analysis, limitations might still exist through combining the age group. Famine exposure has been shown to be associated with adult mortality; thus, survival bias might be possible.

In conclusion, we found that fetal exposure to the Great Chinese Famine significantly increased the risk of CVD in adulthood. However, ICVHMs might modify the effect of famine exposure on CVD risk.

## Data Availability Statement

The original contributions presented in the study are included in the article/Supplementary Material, further inquiries can be directed to the corresponding authors.

## Ethics Statement

The studies involving human participants were reviewed and approved by Ethics Committee of the Kailuan General Hospital. The patients/participants provided their written informed consent to participate in this study.

## Author Contributions

XD, JL, YW, PY, DZ, XY, SC, XL, YL, and SW: writing—original draft. XD, JL, YW, PY, DZ, SC, and XL: investigation. XD, YW, XY, YL, and SW: writing—review and editing. XD, JL, XY, and SW: methodology. SC, XL, YL, and SW: project administration and funding. All authors contributed to the article and approved the submitted version.

## Funding

This study was supported by the Natural Science Foundation of Hebei Province (H2021209018) and Tangshan Science and Technology Innovation Team Program (20130206D).

## Conflict of Interest

The authors declare that the research was conducted in the absence of any commercial or financial relationships that could be construed as a potential conflict of interest.

## Publisher's Note

All claims expressed in this article are solely those of the authors and do not necessarily represent those of their affiliated organizations, or those of the publisher, the editors and the reviewers. Any product that may be evaluated in this article, or claim that may be made by its manufacturer, is not guaranteed or endorsed by the publisher.
