# Supplementary figures and images for: Ideal Cardiovascular Health Metrics Modify the Association Between Exposure to Chinese Famine in Fetal and Cardiovascular Disease: A Prospective Cohort Study
Source: Front Cardiovasc Med. 2021 Nov 4;8:751910. doi: 10.3389/fcvm.2021.751910 (PMC8599955; doi:10.3389/fcvm.2021.751910)

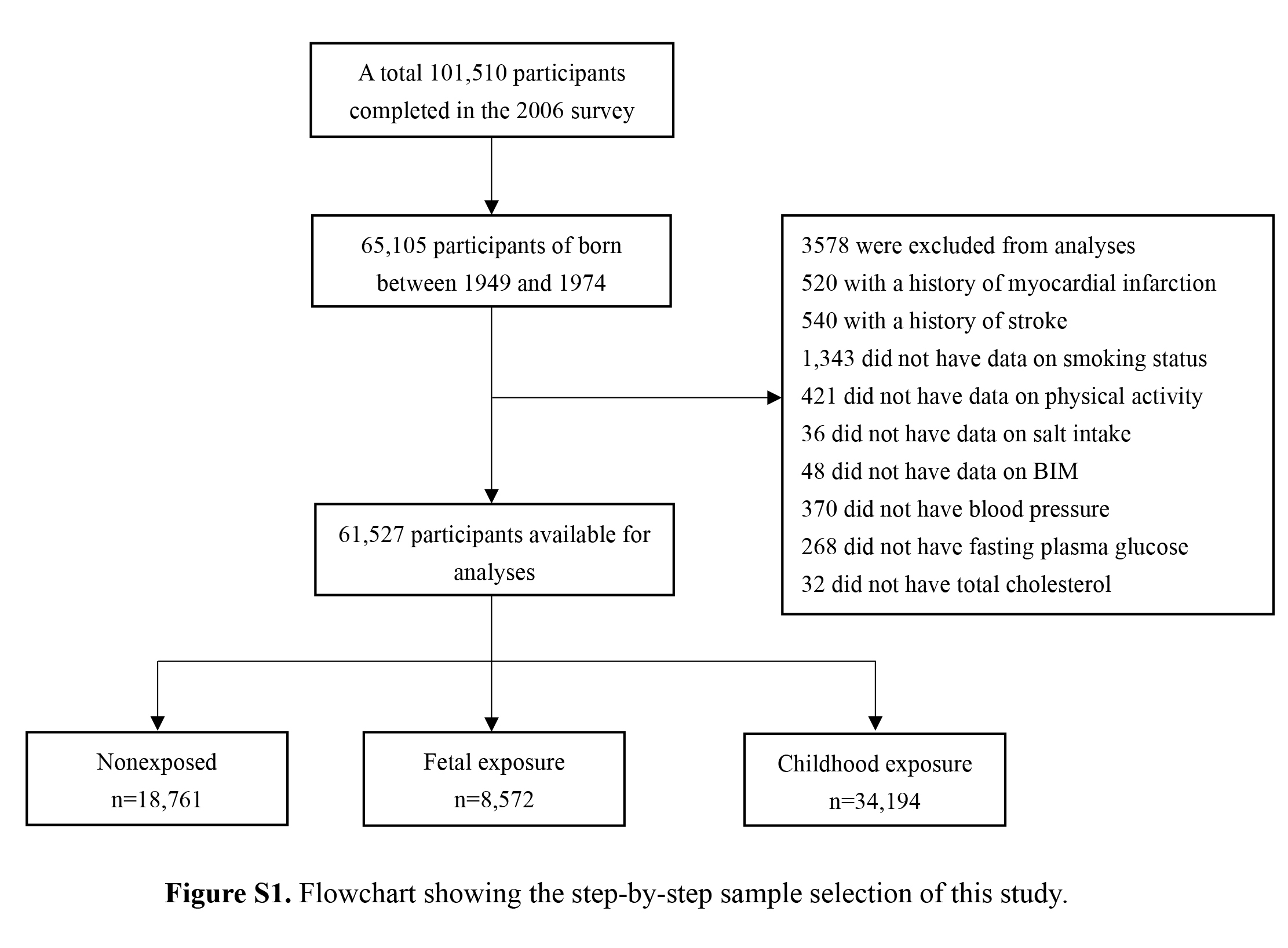

Supplement: Supplementary file 1 [file Image_1.JPEG]
