# Supplementary material for: Ideal Cardiovascular Health Metrics Modify the Association Between Exposure to Chinese Famine in Fetal and Cardiovascular Disease: A Prospective Cohort Study
Source: Front Cardiovasc Med. 2021 Nov 4;8:751910. doi: 10.3389/fcvm.2021.751910 (PMC8599955; doi:10.3389/fcvm.2021.751910)
Supplement: Supplementary file 2 [file Data_Sheet_1.pdf]

Supplementary materials

Table S1. Age-balanced analysis of baseline characteristics of participants according to famine exposure in early life.

	Nonexposed	Fetal	*P* value
Number of participants	52,955	8,572	
Age at baseline, years	48.1±7.0	46.1±1.3	<0.001
Men	41,278(77.9)	6,271(73.2)	<0.001
BMI, kg/m^2	25.1±3.4	25.1±3.4	0.109
High school education or above	9,141(17.3)	1,517(17.7)	0.323
Current drinking	22,040(41.6)	3,508(40.9)	0.225
TC, mmol/L	5.0±1.1	5.0±1.1	0.006
TG, mmol/L	1.3(0.9-2.0)	1.3(0.9-2.0)	0.899
SBP, mmHg	128.9±19.9	127.4±19.1	<0.001
DBP, mmHg	83.8±11.9	83.7±12.0	0.362
FBG, mmol/L	5.5±1.6	5.5±1.7	0.056
Hs-CRP, mg/L	0.7(0.3-1.9)	0.6(0.2-1.6)	<0.001
eGFR, ml/min/1.73m^2	83.1(70.1-98.2)	83.4(70.3-99.2)	0.124
Severity famine exposed	3,476(6.6)	665(7.8)	<0.001
Family history of CVD	3,613(6.8)	603(7.0)	0.472
Use of antihypertensive agent	4,502(8.5)	583(6.8)	<0.001
Use of hypoglycemic medications	887(1.7)	118(1.4)	0.043
Use of lipid-lowering medications	356(0.7)	35(0.4)	0.004
Ideal BP	11,195(21.1)	1,868(21.8)	0.171
Ideal FBG	36,505(68.9)	5,947(69.4)	0.413
Ideal TC	31,578(59.6)	5,006(58.4)	0.031
Ideal BMI	20,354(38.4)	3,364(39.2)	0.154
Ideal smoking	31,440(59.4)	5,129(59.8)	0.418
Ideal salt intake	4,668(8.8)	701(8.2)	0.052
Ideal physical activity	6,004(11.3)	625(7.3)	<0.001
No. of ICVHMs			
≤2	23,715(44.8)	3,968(46.3)	0.002

3-4	25,407(48.0)	4,059(47.4)
≥5	3,833(7.2)	545(6.3)

Abbreviations: BMI, body mass index; TC, total cholesterol; TG, triglycerides; SBP, systolic blood pressure; DBP, diastolic blood pressure; FBG, fasting blood glucose; Hs-CRP, high-sensitivity C-reactive protein; eGFR, estimated glomerular filtration rate; BP, blood pressure; ICVHMs, ideal cardiovascular health metrics.

Data were present as n (%), mean ± SD or median (P_{25}, P_{75}) according to variable category. Pearson's chi-square test, ANOVA analysis or Kruskal-Wallis test were used to compare differences between groups properly.

Table S2. Characteristics of CVD onset according to famine exposure in early life.

	Nonexposed	Famine exposure		*P* value
		Fetal	Childhood	
Number of participants	737	632	3,445	
Age of onset, years	49.5±4.3	54.2±3.8	60.8±4.4	<0.001
Men	670(90.9)	541(85.6)	3,061(88.9)	0.008
ICVHMs				0.143
≤2	445(60.4)	412(65.2)	2,076(60.3)	
3-4	273(37.0)	209(33.1)	1,302(37.8)	
≥5	19(2.6)	11(1.7)	67(1.9)	

Abbreviations: CVD, cardiovascular disease; ICVHMs, ideal cardiovascular health metrics.

Data were present as n (%), mean ± SD or median (P_{25}, P_{75}) according to variable category. Pearson's chi-square test, ANOVA analysis or Kruskal-Wallis test were used to compare differences between groups properly.

Table S3. HR (95% CI) for incident CVD according to famine exposure in early life by age-balanced analysis.

	Nonexposed	Fetal famine exposure
Case subjects/total number	4,182/52,955	632/8,572
IR, 1000 person-years	6.42(6.22-6.61)	5.95(5.51-6.44)
Univariate model	1.00(Reference)	0.93(0.85-1.01)
Age- and sex- adjusted model	1.00(Reference)	1.23(1.13-1.34)
Multivariate model 1	1.00(Reference)	1.24(1.13-1.35)
Multivariate model 2	1.00(Reference)	1.21(1.11-1.32)

Abbreviations: HR, hazard ratio; CI, confidence interval; CVD, cardiovascular disease; IR, incidence rate.

Multivariate model 1: Adjusted for age, sex, education attainment (less than high school, high school or above), drinking (current, never/former), Hs-CRP (< 1.0, 1.0 ≤ Hs-CRP≤ 3.0, or > 3.0 mg/L), eGFR (< 30, 30 ≤eGFR< 60, or ≥60 ml/min/1.73m^2), TG (< 1.7, 1.7 ≤TG< 2.3, or ≥ 2.3 mmol/L).
Multivariate model 2: Included covariates in multivariate model 1 and further adjusted for the severity famine exposed, family history of CVD, use of antihypertensive, hypoglycemic, and lipid-lowering medications (yes/no for each), and individual ideal cardiovascular health metrics.

Table S4. Age-balanced analysis of the number of ICVHMs influence on famine exposed and CVD risk.

	Case subjects/total number	IR, 1000 person-years	Nonexposed	Fetal famine exposure
No. of ICVHMs				
≤2	2,933/27,683	8.73(8.42-9.05)	1.00(Reference)	1.22(1.09-1.36)
3-4	1,784/29,466	4.87(4.65-5.10)	1.00(Reference)	1.18(1.01-1.37)
≥5	97/4,378	1.75(1.43-2.13)	1.00(Reference)	1.25(0.65-2.41)

Abbreviations: CVD, cardiovascular disease. IR, incidence rate; ICVHMs, ideal cardiovascular health metrics.
Adjusted for age, sex, education attainment (less than high school, high school or above), drinking (current, never/former), Hs-CRP (< 1.0, 1.0 ≤ Hs-CRP≤ 3.0, or > 3.0 mg/L), eGFR (< 30, 30 ≤eGFR< 60, or ≥60 ml/min/1.73m^2), TG (< 1.7, 1.7 ≤TG< 2.3, or ≥ 2.3 mmol/L), severity famine exposed, family history of CVD, use of antihypertensive, hypoglycemic, and lipid-lowering medications (yes/no for each).

Table S5. Multivariable adjusted HR (95% CI) for incident CVD according to famine exposure and combined ICVHMs by multiple testing via FDR.

	Case subjects/total number	IR, 1000 person-years	Nonexposed	Fetal famine exposure		Childhood famine exposure	
				HR (95% CI)	*P* value	HR (95% CI)	*P* value
BP							
Ideal	473/13,063	2.87(2.62-3.14)	1.00(Reference)	1.31(0.91-1.91)	0.150	1.01(0.61-1.67)	0.963
Nonideal	4,341/48,464	7.32(7.10-7.54)	1.00(Reference)	1.21(1.06-1.37)	0.005*	1.01(0.85-1.19)	0.904
FBG							
Ideal	2,832/42,452	5.37(5.18-5.57)	1.00(Reference)	1.17(0.99-1.37)	0.058	0.98(0.80-1.20)	0.833
Nonideal	1,982/19,075	8.58(8.21-8.97)	1.00(Reference)	1.31(1.08-1.60)	0.006*	1.08(0.84-1.39)	0.544
TC							
Ideal	2,457/36,584	5.42(5.21-5.64)	1.00(Reference)	1.26(1.06-1.50)	0.008*	1.02(0.82-1.28)	0.847

Nonideal	2,357/24,943	7.73(7.42-8.05)	1.00(Reference)	1.18(0.99-1.40)	0.071	1.01(0.81-1.27)	0.923
BMI							
Ideal	1,353/23,718	4.59(4.35-4.84)	1.00(Reference)	1.11(0.88-1.41)	0.372	0.92(0.68-1.25)	0.596
Nonideal	3,461/37,809	7.47(7.22-7.72)	1.00(Reference)	1.28(1.10-1.47)	0.001*	1.06(0.88-1.28)	0.535
Smoking							
Ideal	2,495/36,569	5.51(5.30-5.73)	1.00(Reference)	1.22(1.03-1.45)	0.025*	0.94(0.75-1.18)	0.608
Nonideal	2,319/24,958	7.59(7.29-7.91)	1.00(Reference)	1.24(1.04-1.48)	0.015*	1.11(0.89-1.39)	0.360
Salt intake							
Ideal	395/5,369	5.93(5.38-6.55)	1.00(Reference)	1.15(0.73-1.79)	0.549	1.11(0.63-1.94)	0.714
Nonideal	4,419/56,158	6.39(6.21-6.58)	1.00(Reference)	1.23(1.08-1.40)	0.002*	1.01(0.86-1.19)	0.915
Physical activity							
Ideal	563/6,629	6.91(6.36-7.51)	1.00(Reference)	1.10(0.69-1.74)	0.691	0.95(0.55-1.64)	0.849
Nonideal	4,251/54,898	6.28(6.10-6.47)	1.00(Reference)	1.23(1.08-1.40)	0.002*	1.01(0.86-1.20)	0.884

Abbreviations: HR, hazard ratio; CI, confidence interval; CVD, cardiovascular disease; ICVHMs, ideal cardiovascular health metrics; FDR, false discovery rate; IR, incidence rate; BP, blood pressure; FBG, fasting blood glucose; TC, total cholesterol; BMI, body mass index.

Adjusted for age, sex, education attainment (less than high school, high school or above), drinking (current, never/former), Hs-CRP (< 1.0, 1.0 ≤ Hs-CRP≤ 3.0, or > 3.0 mg/L), eGFR (< 30, 30 ≤eGFR< 60, or ≥60 ml/min/1.73m^2), TG (< 1.7, 1.7 ≤TG< 2.3, or ≥ 2.3 mmol/L), severity famine exposed, family history of CVD, use of antihypertensive, hypoglycemic, and lipid-lowering medications (yes/no for each), and individual ideal cardiovascular health metrics were mutually adjusted.

* FDR<0.05.

Table S6. Age-balanced analysis of the ICVHMs' influence on famine exposure and CVD risk.

	Case subjects/total number	IR, 1000 person-years	Nonexposed	Fetal famine exposure
BP				
Ideal	473/13,063	2.87(2.62-3.14)	1.00(Reference)	1.31(1.01-1.69)
Nonideal	4,341/48,464	7.32(7.10-7.54)	1.00(Reference)	1.20(1.09-1.32)
FBG				
Ideal	2,832/42,452	5.37(5.18-5.57)	1.00(Reference)	1.18(1.05-1.32)
Nonideal	1,982/19,075	8.58(8.21-8.97)	1.00(Reference)	1.26(1.10-1.44)
TC				
Ideal	2,457/36,584	5.42(5.21-5.64)	1.00(Reference)	1.25 (1.10-1.41)
Nonideal	2,357/24,943	7.73(7.42-8.05)	1.00(Reference)	1.17(1.03-1.32)

BMI				
Ideal	1,353/23,718	4.59(4.35-4.84)	1.00(Reference)	1.17(0.98-1.38)
Nonideal	3,461/37,809	7.47(7.22-7.72)	1.00(Reference)	1.23(1.12-1.37)
Smoking				
Ideal	2,495/36,569	5.51(5.30-5.73)	1.00(Reference)	1.26(1.11-1.42)
Nonideal	2,319/24,958	7.59(7.29-7.91)	1.00(Reference)	1.17(1.04-1.33)
Salt intake				
Ideal	395/5,369	5.93(5.38-6.55)	1.00(Reference)	1.08(0.78-1.49)
Nonideal	4,419/56,158	6.39(6.21-6.58)	1.00(Reference)	1.22(1.12-1.34)
Physical activity				
Ideal	563/6,629	6.91(6.36-7.51)	1.00(Reference)	1.13(0.81-1.58)
Nonideal	4,251/54,898	6.28(6.10-6.47)	1.00(Reference)	1.22(1.11-1.34)

Abbreviations: CVD, cardiovascular disease; IR, incidence rate; BP, blood pressure; FBG, fasting blood glucose; TC, total cholesterol; BMI, body mass index.

Adjusted for age, sex, education attainment (less than high school, high school or above), drinking (current, never/former), Hs-CRP (< 1.0, 1.0 ≤ Hs-CRP≤ 3.0, or > 3.0 mg/L), eGFR (< 30, 30 ≤eGFR< 60, or ≥60 ml/min/1.73m^2), TG (< 1.7, 1.7 ≤TG< 2.3, or ≥ 2.3 mmol/L), severity famine exposed, family history of CVD, use of antihypertensive, hypoglycemic, and lipid-lowering medications (yes/no for each), and individual ideal cardiovascular health metrics were mutually adjusted.
